# Supplementary material for: The cellular environment shapes the nuclear pore complex architecture
Source: Nature. 2021 Oct 13;598(7882):667–71. doi: 10.1038/s41586-021-03985-3 (PMC8550940; doi:10.1038/s41586-021-03985-3)
Supplement: Supplementary file 1 — Reporting Summary [file 41586_2021_3985_MOESM1_ESM.pdf]

nature portfolio

Corresponding author(s): Medalia, O. and Schwartz, T.U.

Last updated by author(s): Aug 16, 2021

# Reporting Summary

Nature Portfolio wishes to improve the reproducibility of the work that we publish. This form provides structure for consistency and transparency in reporting. For further information on Nature Portfolio policies, see our Editorial Policies and the Editorial Policy Checklist.

## Statistics

For all statistical analyses, confirm that the following items are present in the figure legend, table legend, main text, or Methods section.

| n/a | Confirmed | |
|---|---|---|
| ☐ | ☒ | The exact sample size (*n*) for each experimental group/condition, given as a discrete number and unit of measurement |
| ☐ | ☒ | A statement on whether measurements were taken from distinct samples or whether the same sample was measured repeatedly |
| ☐ | ☒ | The statistical test(s) used AND whether they are one- or two-sided<br>*Only common tests should be described solely by name; describe more complex techniques in the Methods section.* |
| ☒ | ☐ | A description of all covariates tested |
| ☒ | ☐ | A description of any assumptions or corrections, such as tests of normality and adjustment for multiple comparisons |
| ☒ | ☐ | A full description of the statistical parameters including central tendency (e.g. means) or other basic estimates (e.g. regression coefficient) AND variation (e.g. standard deviation) or associated estimates of uncertainty (e.g. confidence intervals) |
| ☐ | ☒ | For null hypothesis testing, the test statistic (e.g. *F*, *t*, *r*) with confidence intervals, effect sizes, degrees of freedom and *P* value noted<br>*Give P values as exact values whenever suitable.* |
| ☒ | ☐ | For Bayesian analysis, information on the choice of priors and Markov chain Monte Carlo settings |
| ☒ | ☐ | For hierarchical and complex designs, identification of the appropriate level for tests and full reporting of outcomes |
| ☒ | ☐ | Estimates of effect sizes (e.g. Cohen's *d*, Pearson's *r*), indicating how they were calculated |

*Our web collection on statistics for biologists contains articles on many of the points above.*

## Software and code

Policy information about availability of computer code

| | |
|---|---|
| Data collection | Tomo v5.3.0 (ThermoFisher) for operation of electron microscope (referenced in Methods) |
| Data analysis | Cryo-ET data processing (all available and referenced in Methods): IMOD v4.11.4 (open source, cited), TOM toolbox (open source, cited), Relion v3.1.2_cu9.2 (open source, cited), MATLAB R2019a (MathWorks)<br>Modeling/visualization: Chimera v1.15rc (open source, cited), IMOD v4.11.4 (open source, cited), TOM toolbox v2008 (open source, cited)<br>Statistics/data visualization: Python v3.8.8, Matplotlib v3.3.4, SciPy v1.6.2, StatsModels v0.12.2, Prism v9 (Graphpad) |

For manuscripts utilizing custom algorithms or software that are central to the research but not yet described in published literature, software must be made available to editors and reviewers. We strongly encourage code deposition in a community repository (e.g. GitHub). See the Nature Portfolio guidelines for submitting code & software for further information.

## Data

Policy information about availability of data

All manuscripts must include a data availability statement. This statement should provide the following information, where applicable:

- Accession codes, unique identifiers, or web links for publicly available datasets
- A description of any restrictions on data availability
- For clinical datasets or third party data, please ensure that the statement adheres to our policy

Cryo-EM maps for the human DLD-1 NPC have been deposited in the Electron Microscopy Data Bank (EMDB) with the following accession codes: EMD-12811 (CR), EMD-12812 (IR), EMD-12813 (NR), and EMD-12814 (full composite NPC). Coordinate files for the CR, IR, and NR docked complexes have been deposited in the Protein Data Bank (PDB) with the following accession codes: PDB-7PEQ (CR and NR complex) and PDB-7PER (IR complex). Representative tilt series of lamella from DLD-1 cells have been deposited in the EMDB under accession code EMPIAR-10700 (wild-type, non-depleted cells) and EMPIAR-10701 (Nup96-depleted cells).

# Field-specific reporting

Please select the one below that is the best fit for your research. If you are not sure, read the appropriate sections before making your selection.

☒ Life sciences ☐ Behavioural & social sciences ☐ Ecological, evolutionary & environmental sciences

For a reference copy of the document with all sections, see nature.com/documents/nr-reporting-summary-flat.pdf

# Life sciences study design

All studies must disclose on these points even when the disclosure is negative.

| | |
|---|---|
| Sample size | Sample sizes were determined by available cryo-electron microscopy and cryo-FIB instrument time. For the wild-type NPC structure, 194 NPCs were extracted from 54 tomograms. For Nup96-depleted NPC structures, 163 NPCs were extracted from 71 tomograms. The sample size is sufficient to obtain a structure at the reported resolution. |
| Data exclusions | For cryo-ET: Tomograms exhibiting errors during data collection or lacking NPCs were excluded. During subtomogram averaging and alignment some incomplete NPC subvolumes were manually removed (described in Methods). Exclusion of error-containing or incomplete data is a standard, pre-established practice for cryo-ET processing.<br><br>For NPC diameter measurements: When an accurate measurement was not possible because of strong misalignment or poor signal to noise, no diameter was measured. Sample size for diameter measurements is included with each plot. |
| Replication | Tomograms were acquired from multiple cell samples in each condition and thus served as biological replicates. We have included a statement of reproducibility in regards to the representative micrographs in the manuscript. Representative micrographs are provided in Fig. 1a and Extended Figs. 6a, 6f. The micrograph in Fig. 1a highlights the 3-ring NPC architecture directly visualized in cryo-ET and was chosen from the dataset of 54 wild-type DLD-1 cell tomograms, all of which reproducibly show a 3-ring architecture. The micrograph in Extended Data Fig. 6a was chosen from the auxin-depleted DLD-1 cell dataset (71 tomograms), and this image specifically chosen because it highlights single-ring NPCs that we describe in this manuscript. Finally, the micrograph in Extended Data Fig. 6f was specifically chosen to provide the reader with an anecdotal observation we only identified twice in the dataset (2 out of 73 tomograms) and these tomograms were excluded from subsequent processing (71 tomograms used for subtomogram averaging). |
| Randomization | Cells for cryo-FIB milling and tomogram collection were chosen at random on each TEM grid. Division of the two half-sets of data used for resolution estimation was based on the tomogram number being even or odd. |
| Blinding | Blinding was performed during sample preparation and data collection as cellular samples were given a unique identification number and only de-identified afterward for classification and data processing procedures. |

# Reporting for specific materials, systems and methods

We require information from authors about some types of materials, experimental systems and methods used in many studies. Here, indicate whether each material, system or method listed is relevant to your study. If you are not sure if a list item applies to your research, read the appropriate section before selecting a response.

## Materials & experimental systems

| n/a | Involved in the study | |
|---|---|---|
| ☒ | ☐ | Antibodies |
| ☐ | ☒ | Eukaryotic cell lines |
| ☒ | ☐ | Palaeontology and archaeology |
| ☒ | ☐ | Animals and other organisms |
| ☒ | ☐ | Human research participants |
| ☒ | ☐ | Clinical data |
| ☒ | ☐ | Dual use research of concern |

## Methods

| n/a | Involved in the study | |
|---|---|---|
| ☒ | ☐ | ChIP-seq |
| ☒ | ☐ | Flow cytometry |
| ☒ | ☐ | MRI-based neuroimaging |

## Eukaryotic cell lines

Policy information about cell lines

| | |
|---|---|
| Cell line source(s) | Human colorectal adenocarcinoma cells (DLD-1) with homozygous insertion at Nup96 loci containing NeonGreen moiety and an auxin-inducible degron (Nup96::Neon-AID) from laboratory of M. Dasso (Ref. 23: Regmi et al., 2020). HeLa-derived cell line was created and authenticated in the laboratory of T. Schwartz in an earlier study (Ref. 43: Demircioglu et al., 2019). |
| Authentication | Creation and authentication of the Nup96::Neon-AID DLD-1 cell line was performed in a separate study (Ref. 23: Regmi et al., 2020). Briefly, PCR assays followed by sequencing of PCR products confirmed homozygous insertion of the Neon-AID tag. Subsequently, Western blot analysis confirmed a larger MW protein due to the epitope tag. The HeLa-derived cell line was |

| | |
|---|---|
| | authenticated in a previous study (Ref 43: Demircioglu et al., 2019). |
| Mycoplasma contamination | Cell lines were not tested for Mycoplasma contamination. |
| Commonly misidentified lines<br>(See ICLAC register) | No commonly misidentified cell lines were used in this study. |
